# Transmission of HBV Among Assisted-Living–Facility Residents — Virginia, 2012

**Published:** 2013-05-17

**Authors:** Brooke Rossheim, Stephanie Goodman, Nancy Bryant, Michele Winters-Callender, April Achter, Katie Kurkjian, Mary Beth White-Comstock, Saleem Kamili, Anne Moorman, Melissa Collier

**Affiliations:** Rappahannock Area Health District; Div of Surveillance and Investigation, Virginia Dept of Health; Career Epidemiology Field Officer Program, Office of Public Health Preparedness and Emergency Response; Div of Healthcare Quality Promotion, National Center for Emerging and Zoonotic Diseases; Div of Viral Hepatitis, National Center for HIV/AIDS, Viral Hepatitis, STD, and TB Prevention, CDC

On June 29, 2012, the Rappahannock Area Health District in northwestern Virginia received a report of an acute hepatitis B virus (HBV) infection in an elderly resident of an assisted-living facility (ALF). The resident reported no risk factors for HBV infection except assisted monitoring of blood glucose (AMBG), which has been implicated in the transmission of HBV in ALFs and other long-term–care facilities (1,2). Rappahannock Area Health District investigated the source of the infection and the scope of transmission. Investigators observed facility infection control practices and procedures and conducted staff interviews. The facility was scheduled to close July 31, 2012, necessitating prompt response before residents were transferred.

ALF staff members routinely used pen-shaped lancing devices on multiple residents during AMBG, in contrast with long-standing recommendations and standards of care (3). The Virginia Department of Health (VDH) and CDC recommended testing current residents of the facility. Patient samples were tested for 1) human immunodeficiency virus, 2) hepatitis C virus (HCV) antibody with HCV RNA testing of all positives, 3) HBV DNA, 4) hepatitis B surface antigen, 5) total and immunoglobulin M antibody to hepatitis B core antigen, and 6) antibody to hepatitis B surface antigen. No transmission of human immunodeficiency virus or HCV was identified among the residents. Standard case definitions were used for acute and chronic infection, susceptibility to infection, and immunity (1).

Among current residents, 55 of 59 (93%) were tested, and 17 of 19 (89%) staff members were tested. Among the 55 residents tested, two acutely and two chronically HBV-infected patients were identified; all were aged >60 years and receiving AMBG, none shared rooms. One chronically infected patient transferred from another ALF after being diagnosed with acute HBV infection during an outbreak in January 2011 (1). At the time of the current outbreak, this patient had a high HBV viral load of 6.3×10^10^ IU/mL and appeared to be the source patient because the other chronically infected patient had a very low HBV DNA level. Testing was restricted to residents living at the facility since February 1, 2012, when the apparent source patient was admitted. The remaining three residents who received AMBG were susceptible to HBV infection.

All ALF residents were transferred to new facilities by August 10, 2012. Those facilities were notified by VDH about the outbreak, educated about proper AMBG and infection control practices, and advised to consider patients with diabetes for hepatitis B vaccination based on CDC guidelines (4).

After similar outbreak investigations in Virginia (1,5), VDH has been using intense public health efforts to prevent outbreaks in other facilities. This work has included providing statewide education and training to ALF and nursing home staff members regarding safe AMBG (1,5), developing an infection control toolkit for facilities, and, most recently, partnering with the Virginia Department of Social Services to assess regulatory opportunities. Despite these efforts, HBV transmission occurred subsequently in another Virginia facility as a result of patient transfer. Training ALF and home health agency staff members on the proper methods for AMBG and increased oversight to measure adherence to safe diabetes-care practices remain critical public health priorities to prevent outbreaks of bloodborne pathogens in ALFs.
